# GM-CSF mediates immune evasion via upregulation of PD-L1 expression in extranodal natural killer/T cell lymphoma

**DOI:** 10.1186/s12943-021-01374-y

**Published:** 2021-05-29

**Authors:** Qi-xiang Rong, Fang Wang, Zhi-xing Guo, Yi Hu, Sai-nan An, Min Luo, Hong Zhang, Shao-cong Wu, Hui-qiang Huang, Li-wu Fu

**Affiliations:** State Key Laboratory of Oncology in South China, Collaborative Innovation Center of Cancer Medicine, Sun Yat-sen University Cancer Center, Guangzhou, 510060 Guangdong China

**Keywords:** GM-CSF, PD-L1, JAK/STAT pathway, ENKTL

## Abstract

**Background:**

Granulocyte-macrophage colony stimulating factor (GM-CSF) is a cytokine that is used as an immunopotentiator for anti-tumor therapies in recent years. We found that some of the extranodal natural killer/T cell lymphoma (ENKTL) patients with the treatment of hGM-CSF rapidly experienced disease progression, but the underlying mechanisms remain to be elucidated. Here, we aimed to explore the mechanisms of disease progression triggered by GM-CSF in ENKTL.

**Methods:**

The mouse models bearing EL4 cell tumors were established to investigate the effects of GM-CSF on tumor growth and T cell infiltration and function. Human ENKTL cell lines including NK-YS, SNK-6, and SNT-8 were used to explore the expression of programmed death-ligand 1 (PD-L1) induced by GM-CSF. To further study the mechanisms of disease progression of ENKTL in detail, the mutations and gene expression profile were examined by next-generation sequence (NGS) in the ENKTL patient’s tumor tissue samples.

**Results:**

The mouse-bearing EL4 cell tumor exhibited a faster tumor growth rate and poorer survival in the treatment with GM-CSF alone than in treatment with IgG or the combination of GM-CSF and PD-1 antibody. The PD-L1 expression at mRNA and protein levels was significantly increased in ENKTL cells treated with GM-CSF. *STAT5A* high-frequency mutation including p.R131G, p.D475N, p.F706fs, p.V707E, and p.S710F was found in 12 ENKTL cases with baseline tissue samples. Importantly, *STAT5A-V706fs* mutation tumor cells exhibited increased activation of STAT5A pathway and PD-L1 overexpression in the presence of GM-CSF.

**Conclusions:**

These findings demonstrate that GM-CSF potentially triggers the loss of tumor immune surveillance in ENKTL patients and promotes disease progression, which is associated with STAT5 mutations and JAK2 hyperphosphorylation and then upregulates the expression of PD-L1. These may provide new concepts for GM-CSF application and new strategies for the treatment of ENKTL.

**Supplementary Information:**

The online version contains supplementary material available at 10.1186/s12943-021-01374-y.

## Introduction

ENKTL is a rare type of mature T-cell lymphoma with high malignancy and low sensitivity to anthracycline-based chemotherapy [1–3]. Asparaginase-based chemotherapy combined with radiotherapy is the main treatment for early-stage diseases, while the regimen of treatment for advanced diseases and relapsed/refractory diseases is high-dose chemotherapy followed by autologous hematopoietic stem cell transplant after standard asparaginase-based chemotherapy [4].

Granulocyte-macrophage colony stimulating factor (GM-CSF) is a cytokine that regulates the proliferation and differentiation of myeloid stem cells and mature granulocytes [5]. Recombinant human GM-CSF (hGM-CSF) was used to prevent and treat leukopenia, bone marrow hematopoietic dysfunction, myelodysplastic syndrome [6, 7], and is used as an immunopotentiator for anti-tumor therapies in recent years [8–10]. The biological effects of GM-CSF primarily depend on its binding to GM-CSF receptor α (GM-CSFRα). Activation of the GM-CSF receptor is known to stimulate the janus kinase/signal transducer and activator of transcription (JAK/STAT) pathway, which has proved to be one of the pathways that regulate the expression of PD-L1 [11, 12].

PD-1/CD279 is one of the co-inhibitory receptors that expresses on antigen-stimulated T-cells and interacts with two ligands, PD-L1 and PD-L2 [13]. Elevated expression of PD-L1 is related to poor prognosis and chemotherapy resistance, which has been confirmed in several types of tumors, including ENKTL [14, 15]. Immunotherapies targeting PD1/PD-L1 have shown potential efficacy in a wide range of tumors, such as melanoma, non-small cell lung cancer (NSCLC), renal cell carcinoma, and Hodgkin’s lymphoma [16–19]. Remarkably, it has been reported that PD-1 blockade was a potent strategy for NK/T-cell lymphomas when L-asparaginase regimens fail [20].

From clinical observation, we found that some of the ENKTL patients would develop disease progression after GM-CSF treatment. A recent study has reported that gastric cancer-derived GM-CSF activated neutrophils and induced neutrophil PD-L1 expression via JAK2/STAT3 signaling pathway. And the activated PD-L1 positive neutrophils effectively suppressed normal T-cell immunity in vitro and contributed to the growth and progression in gastric cancer in vivo [21]. Owing to the importance of JAK2/STAT pathway in hematological malignancies [22, 23], and recurrent aberrations of JAK2 in several types of lymphoma, especially T-cell lymphoma [24], we hypothesized that GM-CSF might induce disease progression of ENKTL via JAK/STAT/PD-L1 axis.

In the current study, we found the disease progression caused by GM-CSF in T-cell lymphoma was associated with the decreases of tumor-infiltrating T cells and granzyme B release, which could be restored with PD-1 therapy. JAK2 hyperphosphorylation was related to poor survival and disease progression induced by GM-CSF. According to the next-generation sequencing (NGS) results, *STAT5A* was identified as one of the most frequently mutated genes in ENKTL patients. *STAT5A* mutations resulted in constitutive activation of STAT5 and PD-L1 expression. GM-CSF treatment induced higher PD-L1 expression in *STAT5A* mutated cells than in *STAT5A* wild-type cells. Together, these findings demonstrated that STAT5A mutations and JAK2 hyperphosphorylation might be the trigger for upregulation of PD-L1 induced by GM-CSF in ENKTL cells. It might explain the reason why ENKTL patients commonly underwent disease progression after GM-CSF treatment.

## Materials and methods

### Cell lines and cell culture conditions

SNK-6, SNT-8, and NK-92 cell lines were purchased from BeNa Culture Collection (BNCC), NK-YS cell line was provided by Dr. Wenqi Jiang (Sun Yat-sen University Cancer Center), EL4 and 293 T cell lines were obtained from the American Type Culture Collection (ATCC, USA). All cell lines were validated by short-tandem-repeat (STR). NK-YS, SNK-6, and SNT-8 cells were cultured in RPMI-1640 and IL-2 (100 IU/ml, for NK-YS, SNK-6, and SNT-8 cells) and 293 T cells were grown in DMEM medium containing 10% fetal bovine serum. EL4 cells were cultured in DMEM with 10% fetal equine serum. NK-92 cells were grown in 75% alpha-MEM supplemented with 12.5% fetal bovine serum, 12.5% horse serum, 2 mM l-glutamine, 10 mL/L penicillin-streptomycin, 10 ng/mL recombinant human IL-2 (rIL-2), and 5 mg/mL plasmocin to prevent mycoplasma contamination. All cells were maintained at 37 °C in a humidified 5% CO2 incubator [25, 26].

### Cell viability assays

NK-YS, SNK-6, and SNT-8 cells were treated with 0, 100 and 500 ng/ml Recombinant Human GM-CSF Protein (R&D Systems, USA) for 0, 24, 48, 72 h. For Fedratinib treatment assays, NK-YS, SNK-6, and SNT-8 cells were treated with DMSO or Fedratinib (MedChemExpress, MCE)) at a concentration of 3 nM for 12 h. Cell viability was determined by CCK-8 (Yeasen Biotech, China) [27].

### Western blot analysis

Cells were treated with the indicated concentrations as shown in the figures and washed twice with cold PBS. Whole-cell extracts were collected in RIPA lysis buffer (Santa Cruz Biotechnology, Germany), and protein concentration of the lysates was measured using a BCA Protein Assay Kit (Pierce Biotechnology, USA). The protein samples were electrophoresed through a 10% SDS-PAGE gel and transferred to a polyvinylidene difluoride (PVDF) membrane (Millipore, USA). After blocking, membranes were probed with primary antibodies (1:1000) followed by washing and incubation with a secondary antibody (1:5000) conjugated to horseradish peroxidase (Amersham GE Healthcare, USA). Protein bands were visualized by applying a chemiluminescent reagent (Pierce ECL kit, Thermo Fisher Scientific, USA) [28]. The antibody against Phospho-Jak2 (Tyr1007/1008), JAK2, Phospho-Stat5 (Tyr694), STAT5, PD-L1 were purchased from Cell Signaling Technology (USA). The antibody against Phospho-STAT5A (Y694), STAT5A, Phospho-STAT5B (S731), STAT5B were purchased from Abcam (USA).

### Dual-luciferase reporter assay

The template of PD-L1 (Accession: NM_001267706) promoter fragment was purchased from GeneCopoeia Inc. (# HPRM40139) and was amplified into the promoter-less plasmid pGL3-Basic vector by PCR (Promega, USA). The *STAT5A* and *STAT5B* fragments were amplified using the relevant primers by PCR and inserted into the pReceiver-M12 vector (GeneCopoeia Inc., USA). When reaching an approximately 80% confluence, 4 × 10^5^ 293 T cells each were co-transfected with 3.8 μg/well of pGL3 luciferase construct (empty vector or pGL3-PD-L1 promoter) and 0.2 μg/well pRL-TK (Promega, USA). The relative luciferase activity was examined by Dual Luciferase Assay Kit (Promega, USA) following the manufacturer’s protocols [29].

### RNA extraction and quantitative real-time PCR

Total cellular RNA was isolated using Trizol (Life Technologies, USA) according to the manufacturer’s protocol. For first-strand cDNA synthesis, 5μg of total RNA was reverse-transcribed using the GoScript™ Reverse Transcription System kit (Promega, USA) followed by quantitative polymerase chain reaction (qPCR) with GoTaq qPCR Master Mix (Promega, USA), according to the manufacturer’s instructions. Real-time PCR analyses were conducted using the Biorad CFX96 system with SYBR green (Bio-Rad, USA) and the appropriate primers to estimate the mRNA expression levels of PD-L1. The primers are as follows: PDL1 forward: 5′-TATGGTGGTGCCGACTACAA-3′; reverse: 5′-TGCTTGTCCAGATGACTTCG-3′; GAPDH forward: 5′-CTCCTCCTGTTCGACAGTCAGC-3′; reverse: 5′-CCCAATACGACCAAATCCGTT-3′. Data were normalized to GAPDH levels. Experiments were performed in triplicates [30].

### Transfection of shRNAs and plasmid DNAs

*STAT5A* and *STAT5B* shRNAs and an shRNA scramble control (Open Biosystems GE Healthcare Dharmacon Inc., USA) were transiently transfected along with a pSIH-H1-puro Lentivector Packaging Kit (System Biosciences, USA). Transfections were carried out in 293 T cells reaching ∼80% confluency in 10 cm dishes using Lipofectamine 2000 transfection reagent (Life Technologies, USA) and following the manufacturer’s instructions. Transfection medium was replaced with fresh growth medium 5 h after transfection. At 48 h following the initial transfection, viral supernatant was collected and filtered through a 0.45 nm filter (System Biosciences, USA). SNK-6 and SNT-8 cells were infected and incubated with the viral particles overnight at 37 °C. At 48 h after transfection, cells were placed under puromycin selection by supplementing the growth medium with puromycin (3 μg/ml for SNK-6, and 5μg/ml for SNT-8). Growth medium was replaced every 48 h for 2–3 weeks until isolated colonies (∼2 mm diameter) were apparent on the plate. At this point, individual clones were transferred to 12-well dishes and expanded in 1 μg/ml puromycin for further analysis. Individual clones were verified by Western blot and RT-PCR [31].

### In vivo mouse studies

C57BL/6 mouse was purchased from Beijing Vital River Laboratory Animal Technology Co., Ltd., and kept in a specific pathogen-free (SPF) barrier facility at the Animal Center of Sun Yat-sen University Cancer Center (*n* = 80). 8–12 weeks old female mice were used for all animal experiments. Experiments were approved by the institutional committee of Sun Yat-sen University Cancer Center (No. L102042018120I), and conducted following protocols approved by the Guangdong Provincial Animal Care and Use Committee.

EL4 cells or B16-F10 cells (5 × 10^5^ cells in 200 μL growth medium) were subcutaneously injected into the right flank of immunocompetent C57BL/6 mouse. Tumor sizes were measured with calipers every 2 days and tumor volumes were calculated by applying the following formula: 1/2 (length×width^2^). When tumor sizes reached approximately 100 mm^3^, mice were randomized into control or experimental groups. The terminal event was defined as tumors reaching a size of 2000 mm^3^ in the control group, at which point animals were euthanized [32, 33].

Mice were intraperitoneally injected with murine GM-CSF (315–03-20; Peprotech; USA) or anti-mouse PD-1 (BE0273; Bio X cell, USA) alone, the combination of murine GM-CSF and anti-mouse PD-1, or saline and rat IgG2a isotype control (BE0089; Bio X cell, USA) (each group, *n* = 8). Murine GM-CSF (9 μg/kg), anti-PD-1 antibody therapy (10 mg/kg), or saline was administered intraperitoneally from day 7, every 3 days, after tumor implantation [34–37]. Survival analysis was performed using Kaplan-Meier analysis and log-rank test.

### Patients and tissue specimens

Clinical data and/or samples of 109 ENKTL patients, 4 peripheral T-cell lymphoma, not otherwise specified (PTCL-NOS) patients, and 4 angioimmunoblastic T-cell lymphoma (AITL) from September 1999 to March 2019 were collected from Sun Yat-sen University Cancer Center (Figs. 6B, 7B), including medical records, tumor samples, peripheral blood, and buccal swabs samples. The clinical characteristics were summarized in Additional file: Table S2. Clinical data were collected from pathology reports and unprocessed medical files. The study was conducted with the permission of the Ethics Committee of the Sun Yat-sen University Cancer Institutional Board, and all patients involved provided informed written consent.

### Histology and immunohistochemistry (IHC)

For IHC staining of the xenografts, tumor tissues were fixed, embedded, and sectioned (3 μm thick). Immunohistochemistry staining for mouse tissues was performed following standard procedures [39]. Two qualified professional pathologists performed a subsequent blinded evaluation. PBS, instead of the primary antibody, was used as a negative control, and specimens were scored according to the intensity of the dye color and the number of positive cells. A staining index (values 0–12), obtained as the product of the intensity of mouse PD-L1, CD3, CD8, granzyme B, and human STAT5, JAK2, PD-L1-positive staining. The intensity of the dye color was graded as 0 (no color), 1 (light yellow), 2 (light brown), or 3 (brown), and the number of positive cells was graded as 0 (< 5%), 1 (5–25%), 2 (25–50%), 3 (51–75%), or 4 (> 75%). The intensity was graded as -, negative staining (0); +, mild expression (1–4); ++, moderate expression (5–8); +++, strong expression (9–12). The following antibodies were used for ENKTL tissues: Anti-Jak2 (phospho Y1007 + Y1008, Abcam, USA), Anti-STAT5 (phosphor Y694, Abcam, USA). The antibody against human PD-L1 was purchased from Cell Signaling Technology (USA). The following antibodies were used for mouse tissues: Anti-CD3 antibody (Abcam, USA), Anti-CD8 antibody (Abcam, USA), Anti-Granzyme B antibody (Abcam, USA). The antibody against mouse PD-L1 was purchased from Cell Signaling Technology (USA) [40].

### Tumor digestion

Tumors were extracted and finely minced. Tumor tissue was additionally blended with Collagenase type IV (Absin, China) for 2 h, 37 °C. Tumors were extracted and re-suspension in PBS buffer containing 2% FBS for flow cytometric analysis.

### Flow cytometry

Tumors were extracted and processed as described above before re-suspension in PBS buffer containing 2% FBS and PBS for flow cytometric analysis. Zombie NIR™ Fixable Viability Kit (Biolegend, USA) was applied to cells for 30 min on ice in the dark. Cells were washed and incubated with fluorochrome-conjugated antibody (anti-mouse CD45 Birliant violet 605, BioLegend; anti-mouse CD3 APC, BioLegend; anti-mouse CD8 FITC, BioLegend) at the manufacturer’s recommended dilution for 30 min on ice in the dark. For samples requiring intracellular staining, cells were fixed with Fixation/Permeablization Diluent (eBioscience cat. 00–5223-56) for 30 min at room temperature, washed twice with Permeablization Buffer (eBioscience cat. 00–8333-56), and incubated with antibody (anti-mouse Granzyme B PE, BioLegend; anti-mouse Ki-67 Alexa Fluor 700, BioLegend) in permeabilization buffer for 30 min at room temperature in the dark. Following staining, cells were washed again with permeabilizaton buffer, subsequently washed with PBS, and re-suspended in PBS buffer for flow cytometric analysis on the CytoFLEX LX Flow Cytometer. 50,000–100,000 cells were analyzed per sample per mouse using Beckman CytExpert Software.

### Next-generation sequencing

Formalin-fixed paraffin-embedded (FFPE) tumor samples and matched peripheral blood or buccal swabs samples of fourteen patients diagnosed with ENKTL were used to extract DNA and detect mutations by targeted next-generation sequencing (NGS) with a panel of the coding sequence of 102 ENKTL-relevant genes (Additional file: Table S1) (GeneseeqOne, Nanjing Geneseeq Technology Inc., China). Sequencing was performed on the Illumina HiSeq4000 platform followed by data analysis as previously described [41].

### Generation and expression of *STAT5A*/*B* constructs

Sanger sequencing was used to identify the *STAT5A*/*B* mutations in ENKTL cell lines, including NK-YS, SNK-6, and SNT-8. Next-generation sequencing (NGS) was used to identify the *STAT5A*/*B* mutations in ENKTL tumor samples. Wild-type *STAT5A* (*STAT5A*^*WT*^) and wild-type *STAT5B* (*STAT5B*^*WT*^) were amplified with PrimeSTAR MAX DNA Polymerase (TaKaRa Bio Inc., code no. R450A) using NK-92 cell line cDNA as the template inserted into the pReceiver-M12 vector. *STAT5A*/*B* mutations were generated from *STAT5A/B*^*WT*^ constructs and confirmed by Sanger sequencing. Eukaryotic expression vectors harboring *STAT5A/B*^*WT*^ or *STAT5A/B* mutations were introduced into NK-92 cells.

### Statistical analysis

Statistical analysis was carried out using IBM SPSS statistics software or GraphPad Prism using Student’s t-test or one-way ANOVA or Dunnett’s test. All experiments were repeated in triplicate. Data are expressed as mean ± standard deviation (SD). Statistical significance was defined as *P* < 0.05.

## Results

### GM-CSF mediates immune evasion and results in tumor progression in ENKTL

**Fig. 1:**
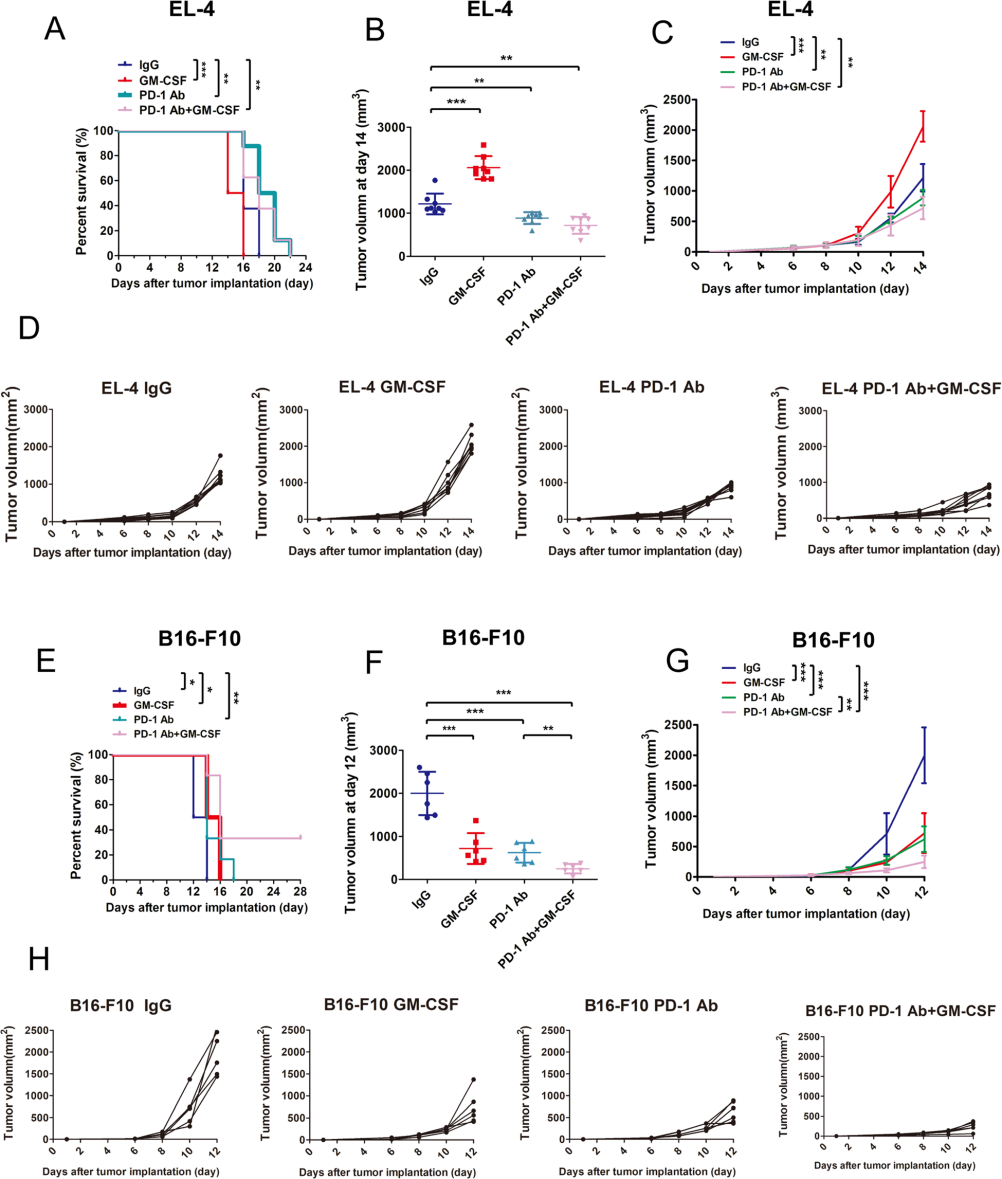
GM-CSF promotes tumor growth in vivo. **a** Survival analysis of C57BL/6 mouse bearing EL4 tumors with different treatments (*n* = 8). **b** Tumor volumes of C57BL/6 mouse bearing EL4 tumors with different treatments at day 14 (n = 8). **c** The mean of tumor volumes determined at the indicated days with different treatments in C57BL/6 mouse bearing EL4 tumors (n = 8). **d** Tumor growth curve of each group of C57BL/6 mouse bearing EL4 tumors with different treatments (n = 8). **e** Survival analysis of C57BL/6 mice bearing B16-F10 tumors with different treatments (*n* = 6). **f** Tumor volumes of C57BL/6 mice bearing B16-F10 tumors with different treatments at day 12 (n = 6). **g** The mean of tumor volumes determined at the indicated days with different treatments in C57BL/6 mice bearing B16-F10 tumors (n = 6). **h** Tumor growth curve of each group of C57BL/6 mice bearing B16-F10 tumors with different treatments (n = 8). Error bars represent the SD of three independent experiments. * *P* < 0.05, ** *P* < 0.01, *** *P* < 0.001

**Fig. 2:**
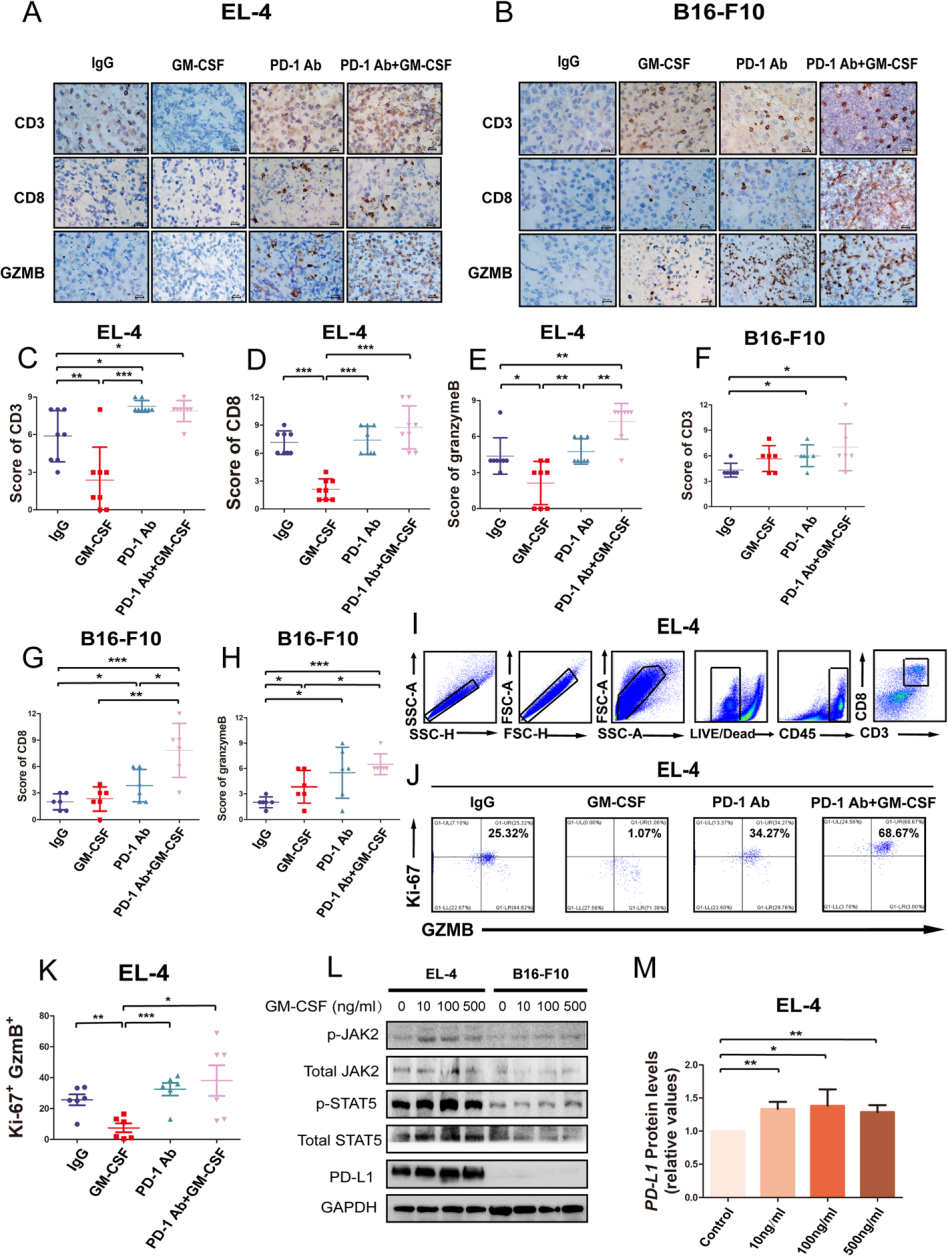
GM-CSF promotes immunosuppression in vivo. **a** The representative immunohistochemical staining of CD3, CD8, and granzyme B in EL4 tumor tissues of C57BL/6 mice was shown. **b** The representative immunohistochemical staining of CD3, CD8, and granzyme B in B16-F10 tumor tissues of C57BL/6 mice were shown. **C** Immunohistochemistry staining of CD3 in tumor tissues of C57BL/6 mouse-bearing EL4 tumors with different treatments (n = 8). **d** Immunohistochemistry staining of CD8 in tumor tissues of C57BL/6 mouse-bearing EL4 tumors with different treatments (n = 8). **e** Immunohistochemistry staining of granzyme B in xenograft tumor tissues of C57BL/6 mouse-bearing EL4 tumors with different treatments (n = 8). **f** Immunohistochemistry staining of CD3 in tumor tissues of C57BL/6 mice bearing B16-F10 tumors with different treatments (n = 6). **g** Immunohistochemistry staining of CD8 in tumor tissues of C57BL/6 mice bearing B16-F10 tumors with different treatments (n = 6). **h** Immunohistochemistry staining of granzyme B in xenograft tumor tissues of C57BL/6 mice bearing B16-F10 tumors with different treatments (n = 8). The staining scores were presented as means ± S. D in the scatter plot. * *P* < 0.05, ** *P* < 0.01, *** *P* < 0.001. **i** Representative contour plots showing the general gating strategy used to identify the purified CD8 T cells (CD45 + CD3 + CD8+) from EL-4 tumors of C57BL/6 with different treatment (n = 6). **j** The percentage of Ki-67 + GzmB+ CD8 T cells of EL-4 tumors of C57BL/6 mouse with different treatment (n = 6). **k** Scatter plots that represent the percentage of Ki-67 + GzmB+ CD8 T cells of EL-4 tumors of C57BL/6 mouse with different treatment (n = 6). **l** EL-4 and B16-F10 cells were treated with GM-CSF (10 ng/ml, 100 ng/ml, and 500 ng/ml) for 12 h, p-JAK2, JAK2, p-STAT5, STAT5, and PD-L1 protein expression was measured by Western blot. **m** Relative protein expression levels of PD-L1 were increased by GM-CSF (10 ng/ml, 100 ng/ml, and 500 ng/ml) treatment in EL-4 and B16-F10 cells

To explore the mechanism of disease progression in vivo*,* we established the mouse models bearing EL4 cell tumors. The results showed that EL4 tumor-bearing mice treated with anti-PD-1 (*n* = 8) or the combined therapy (GM-CSF combined with PD-1 antibody, n = 8) had longer survival (Fig. 1A) and significant delay in tumor growth (Fig. 1B-D) compared with the mice in control group (n = 8) or mice treated with GM-CSF alone (n = 8). To determine the immunological effect of GM-CSF in vivo, we further examined the tumor-infiltrating lymphocytes (TILs) and its activation marker (granzyme B) in tumor tissues derived from mice. Tumors from mice treated with GM-CSF alone have significantly lower levels of CD3, CD8, and granzyme B than tumors from mice treated with IgG control, and tumors from mice received anti-PD-1 therapy or combined therapy have significantly higher levels of CD3 (Fig. 2A, C-E). These results demonstrated that GM-CSF decreased TILs in vivo, which could be reversed by anti-PD-1 therapy. Furthermore, tumors from mice treated with combined therapy have significantly higher granzyme B levels (Fig. 2A, E), indicated that anti-PD-1 therapy combined with GM-CSF might promote the anti-tumor effect of TILs.

We have also established the melanoma mouse model bearing B16-F10 tumors. The results showed that mice bearing B16-F10 tumors treated with combined regimen had longer survival than other groups ((Fig. 1E-H), and tumor samples of mice treated with PD-1 antibody or combined therapy had significantly higher levels of CD3, CD8, and granzyme B than IgG group (Fig. 2F-H). Tumor samples of mice treated by GM-CSF had significantly higher level of granzyme B than IgG group (Fig. 2H).

Then we have tested the expression of CD45, CD3, CD8, ki-67, granzyme B in EL-4 tumor cells of C57BL/6 mice using flow cytometry in another in vivo study, which included 24 C57BL/6 mice bearing EL-4 tumors (Fig. 2I-K). The percentage of Ki-67 + GzmB+ CD8 T cells was significantly lower in tumors of GM-CSF treated mice than IgG group (*n* = 6, *p* = 0.003) (Fig. 2J-K). Ki-67 + GzmB+ CD8 T cells of EL-4 tumors were higher in PD-1 treated (n = 6) and combined regimen treated mice (n = 6), but hasn’t reach the statistical significance (*p* = 0.23 and *p* = 0.08). In addition, the expressions of p-JAK2, p-STAT5, and PD-L1 in EL-4 cells were upregulated after treated with 10 ng/ml, 100 ng/ml, or 500 ng/ml murine GM-CSF for 12 h (Fig. 2L-M).

### GM-CSF upregulates PD-L1 expression in ENKTL via JAK2/STAT5 pathway

**Fig. 3:**
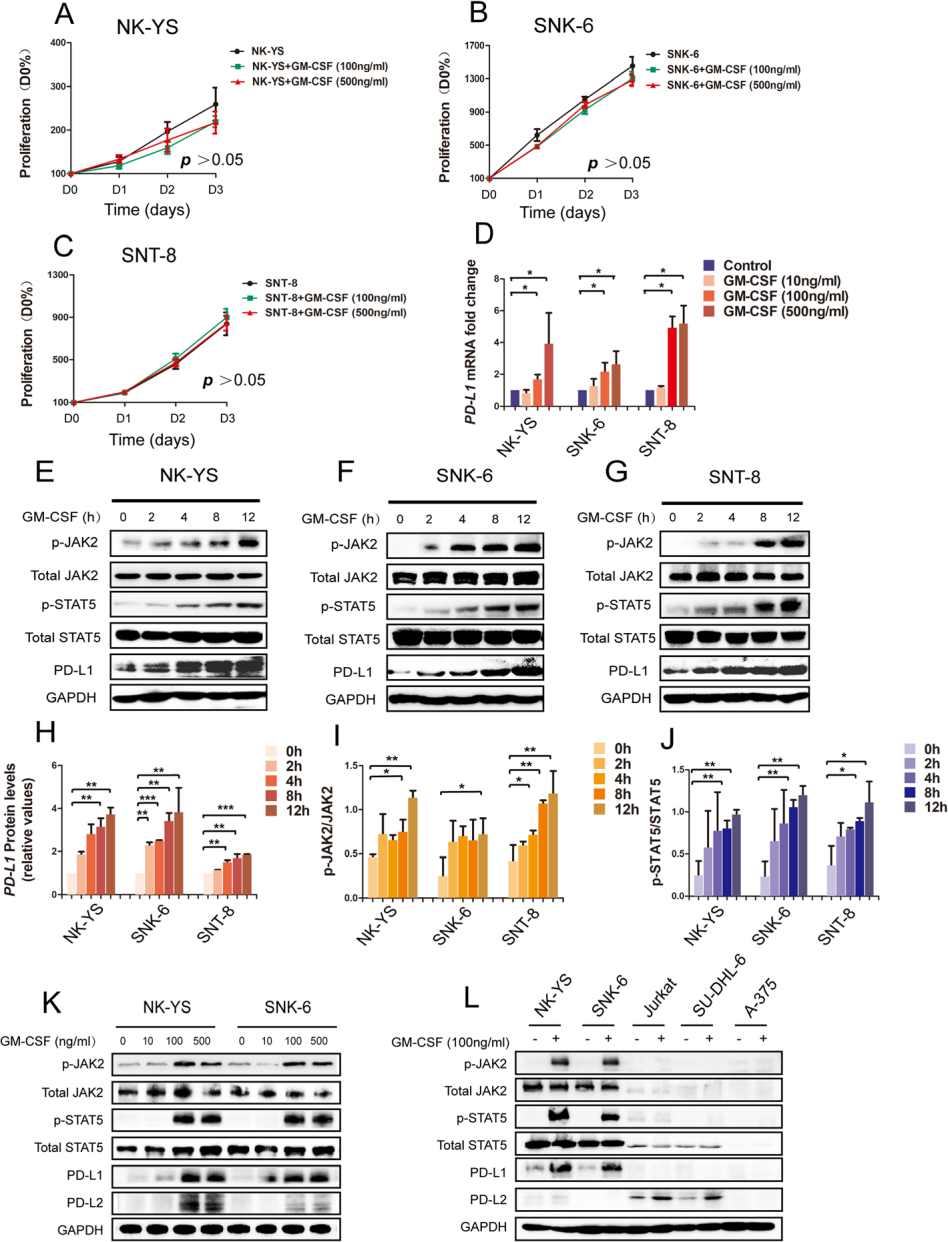
JAK/STAT activation directly drives PD-L1 expression in ENKTL. **a-c** The proliferation of NK-YS, SNK-6, and SNT-8 cells treated by different concentrations of GM-CSF (100 ng/ml and 500 ng/ml). The above assay was determined by CCK8 as described in materials and methods. Each point represents the mean ± standard deviations (SDs) of three independent experiments performed. **d** Relative mRNA expression levels of PD-L1 were increased by GM-CSF (10 ng/ml, 100 ng/ml, and 500 ng/ml) treatment in NK-YS, SNK-6, and SNT-8 cells. **e-g** NK-YS, SNK-6, and SNT-8 cells were treated with GM-CSF (100 ng/ml) for 0, 2, 4, 8, 12 h, p-JAK2, JAK2, p-STAT5, STAT5, and PD-L1 protein expression was measured by Western blot. **d** Relative protein expression levels of PD-L1 were increased by GM-CSF (100 ng/ml) treatment in NK-YS, SNK-6, and SNT-8 cells. **i**, **j** The ratio of protein expression of p-JAK2 to JAK and ratio of protein expression of p-STAT5 to STAT5 in ENKTL cell lines (NK-YS, SNK-6, and SNT-8) treated with GM-CSF (100 ng/ml) for 0, 2, 4, 8, 12 h. **k** ENKTL cell lines NK-YS and SNK-6 were treated by GM-CSF (10 ng/ml, 100 ng/ml, and 500 ng/ml). The protein expressions of p-JAK2, JAK2, p-STAT5, STAT5, and PD-L1 were detected by Western blot. **l** ENKTL cell lines NK-YS and SNK-6, T-cell lymphoblastic lymphoma/leukemia cell line Jurkat, B-cell lymphoma cell line SU-DHL-6, and melanoma cell line A375 were treated by GM-CSF (100 ng/ml). The protein expressions of p-JAK2, JAK2, p-STAT5, STAT5, PD-L1, and PD-L2 were detected by Western blot. * *P* < 0.05, ** *P* < 0.01, *** *P* < 0.001. Error bars represent SD of three independent experiments

To determine the molecular mechanism of GM-CSF-induced disease progression in ENKTL, NK-YS, SNK-6, and SNT-8 cells were treated with 100 ng/ml or 500 ng/ml recombinant human GM-CSF in 0, 24, 48 and 72 h [42, 43]. The cell viability had no statistic differentiation between control and GM-CSF treated groups among all three ENKTL cell lines (Fig. 3A-C), indicating that GM-CSF didn’t affect the proliferation of ENKTL directly. But the mRNA and protein expression levels of PD-L1 strongly increased after GM-CSF (100 ng/ml and 500 ng/ml) treatment in ENKTL cells (Fig. 3D, K). We found that the expressions of p-JAK2, p-STAT5, and PD-L1 were upregulated gradually in the treatment of GM-CSF (100 ng/ml) in a time-dependent manner (Fig. 3E-G), showing that GM-CSF regulated PD-L1 expression in ENKTL via JAK2/STAT5 pathway (Fig. 3H-J). The protein expression levels of PD-L1 in Jurkat, SU-DHL-6, and A-375 cells have no significant change after treated with GM-CSF (Fig. 3L).

**Fig. 4:**
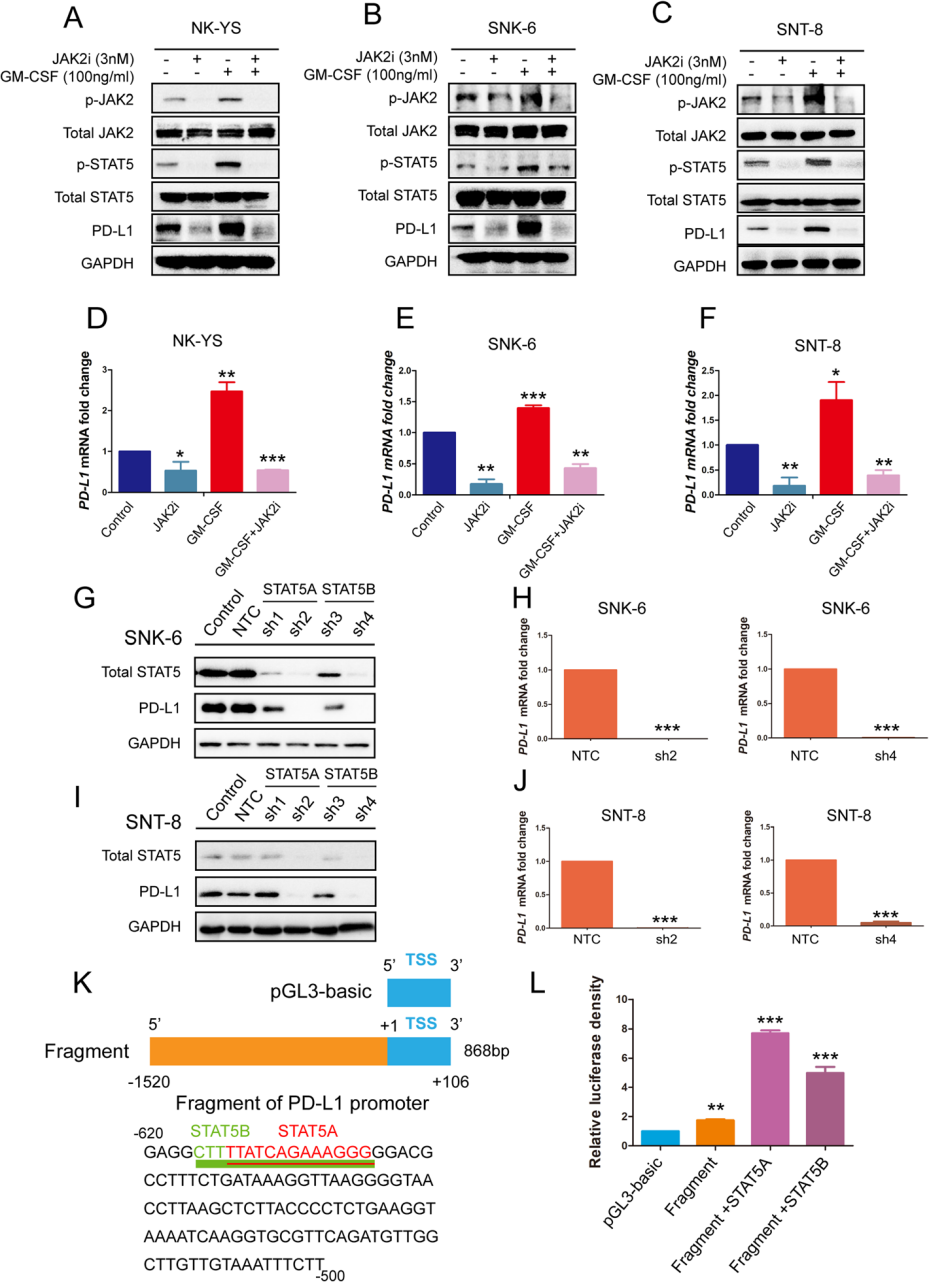
Downregulation of JAK/STAT pathway decreases PD-L1 expression in ENKTL. NK-YS, SNK-6, and SNT-8 cells were treated with JAK2 inhibitor Fedratinib (TG-101348, 3 nM) alone, or GM-CSF (100 ng/ml) alone, or Fedratinib combined with GM-CSF for 12 h. **a-c** Protein expression of p-JAK2, JAK2, p-STAT5, STAT5, and PD-L1 of ENKTL cell lines NK-YS, SNK6, and SNT-8 were measured by Western blot. **d-f** And relative mRNA expression of PD-L1 was measured by quantitative polymerase chain reaction (qPCR). **g-j** SNK-6 and SNT-8 cells expressing shSTAT5 or control were evaluated for STAT5 and PD-L1 protein expression by Western blot, and mRNA expression by qPCR. **k** The − 620 to − 500 nucleotide sequence of the 5′-flanking region of PD-L1 is shown. Underlined sequences are putative STAT5A and STAT5B transcription factor binding sites, as predicted by JASPAR database and PROMO. And PD-L1 promoter fragments cloned into pGL3-Basic vector. **l** Analysis of PD-L1 promoter fragment A constructs in 293 T cells transiently transfected with STAT5A or STAT5B for 48 h. Relative luciferase activity was determined as described. Error bars represent the SD of three independent experiments. * P < 0.05, ** P < 0.01, *** P < 0.001. Error bars represent the SD of three independent experiments

To further confirm the role of JAK2/STAT5 in regulating PD-L1 expression in ENKTL cells, we used the JAK2 inhibitor fedratinib (3 nM) to treat the NK-YS, SNK-6, and SNT-8 cells. The protein levels of p-JAK2, p-STAT5, and PD-L1 were downregulated in these three cells, respectively (Fig. 4A-F). Moreover, the increase of PD-L1 triggered by GM-CSF was impaired with the treatment of fedratinib (Fig. 4A-F), which further proved that GM-CSF regulated the PD-L1 expression in ENKTL via JAK2/STAT5 pathway.

To evaluate whether the genetic depletion of STAT5 could downregulate the expression of PD-L1 in ENKTL cells, *STAT5A* or *STAT5B* was knocked down using four individual short hairpin RNAs (shRNAs) (*STAT5A*: sh1,sh2; *STAT5B*: sh3,sh4) in SNK-6 and SNT-8 cells. Protein levels of PD-L1 were significantly reduced in *STAT5A* and *STAT5B* knockdown cells (Fig. 4G-J). To explore whether transcription factor STAT5A and STAT5B could bind to PD-L1 promoter, we co-transfected pReceiver-M12/*STAT5A* or pReceiver-M12/*STAT5B* plasmids with PD-L1-promoter-Pgl3-Basic vector into 293 T cells. The predicted STAT5A and STAT5B binding sites on PD-L1 promoter were presented using JASPAR databases (http://jaspar.binf.ku.dk/) and PROMO (http://alggen.lsi.upc.es/) (Fig. 4K). In a dual-luciferase reporter assay, we observed that both pReceiver-M12/*STAT5A* and pReceiver-M12/*STAT5B* notably promoted PD-L1 promoter-driven luciferase activity (Fig. 4L).

### JAK2 hyperphosphorylation is correlated with poor prognosis in ENKTL patients treated with GM-CSF

**Fig. 5:**
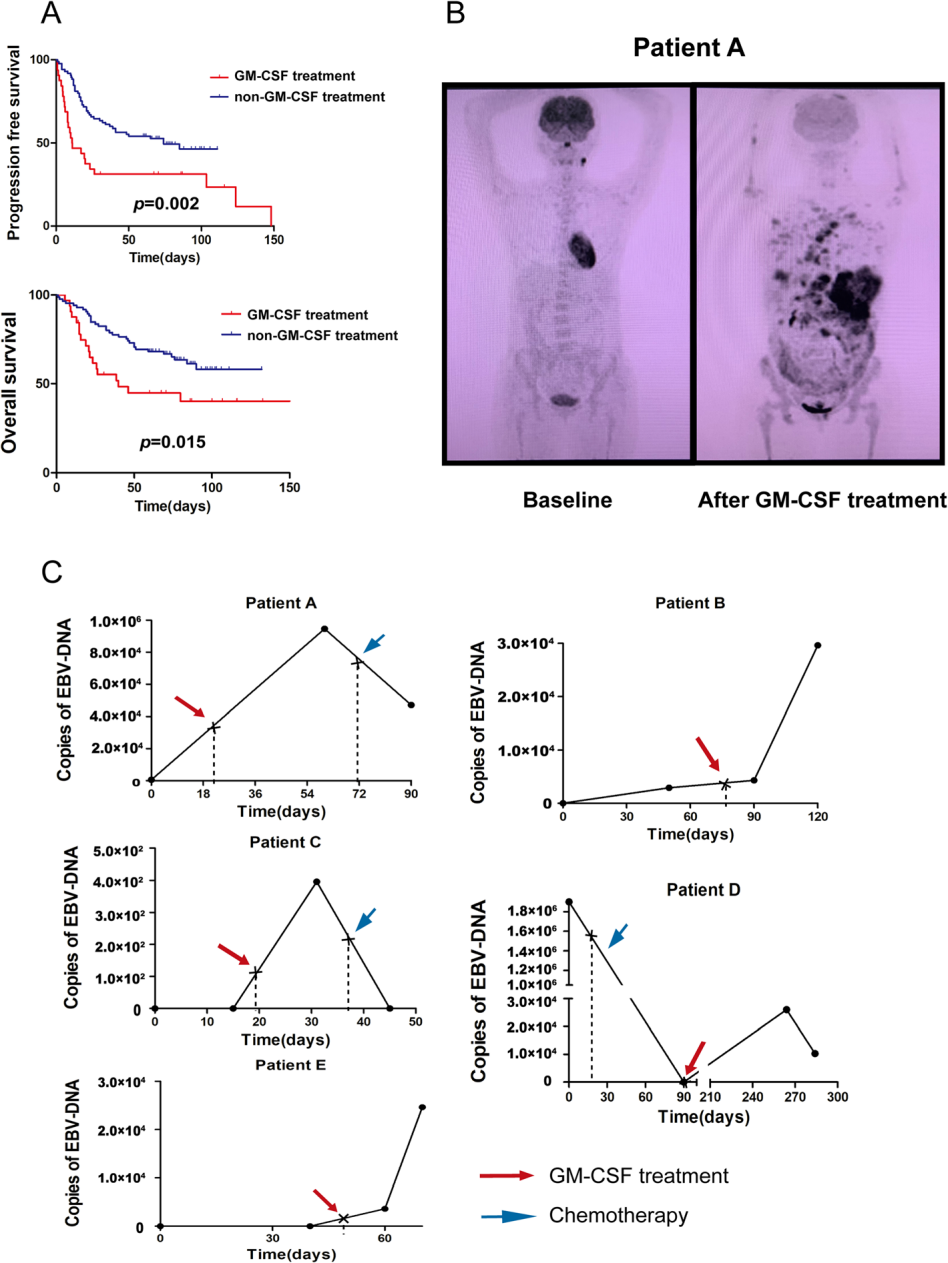
GM-CSF induces disease progression in ENKTL. **a** The PFS and OS of ENKTL patients who have received GM-CSF treatment compared to ENKTL patients who have not received GM-CSF treatment. **b** Imaging data of a patient who had disease progression after treated by GM-CSF. The patient has received good partial remission after chemotherapy and was treated by GM-CSF for hematopoietic stem cell mobilization, who relapsed within 1 month. **c** Test results of serum EBV-DNA of five ENKTL patients who have been treated by GM-CSF. Patients who were treated by GM-CSF could induce elevated serum EBV-DNA copies, which could be reduced after chemotherapy

According to clinical observation, some of the patients with ENKTL developed disease progression after receiving GM-CSF therapy for hematopoietic stem cell mobilization. A total of 117 ENKTL patients were diagnosed in Sun Yat-sen University Cancer Center from September 1999 to April 2017, including 32 patients who have received GM-CSF treatment (1 patient was diagnosed in September 1999, and 31 patients were diagnosed from June 2006 to April 2017), were included in our research. The clinical characteristics were summarized in Additional file: Table S2. After a median follow-up time of 69 months (1–159 months), the 5-year progress-free survival (PFS) and overall survival (OS) were 47.0 and 59.8%. The patients who have received GM-CSF treatment have inferior 5-year PFS (28.1% vs. 54.1%, *p* = 0.002) and 5-year OS (37.5% vs. 68.2%, *p* = 0.015) (Fig. 5A). Univariate analysis revealed that GM-CSF treatment correlated with inferior PFS [*p* = 0.003, HR = 2.16(1.30–3.61)] and OS [*p* = 0.017, HR = 2.03(1.13–3.62)]. Epstein-Barr virus (EBV) infection is the major hypothesis of ENKTL, and EBV-DNA is one of the markers that can monitor the condition of ENKTL [44]. From the 117 ENKTL patients, we have found 5 patients who have comparable imaging data and/or copies of plasma EBV-DNA results during the time of GM-CSF treatment from a retrospective study (Fig. 5B-C). The copies of plasma EBV-DNA of the 5 patients elevated after GM-CSF treatment and decreased after chemotherapy (Fig. 5C).

**Fig. 6:**
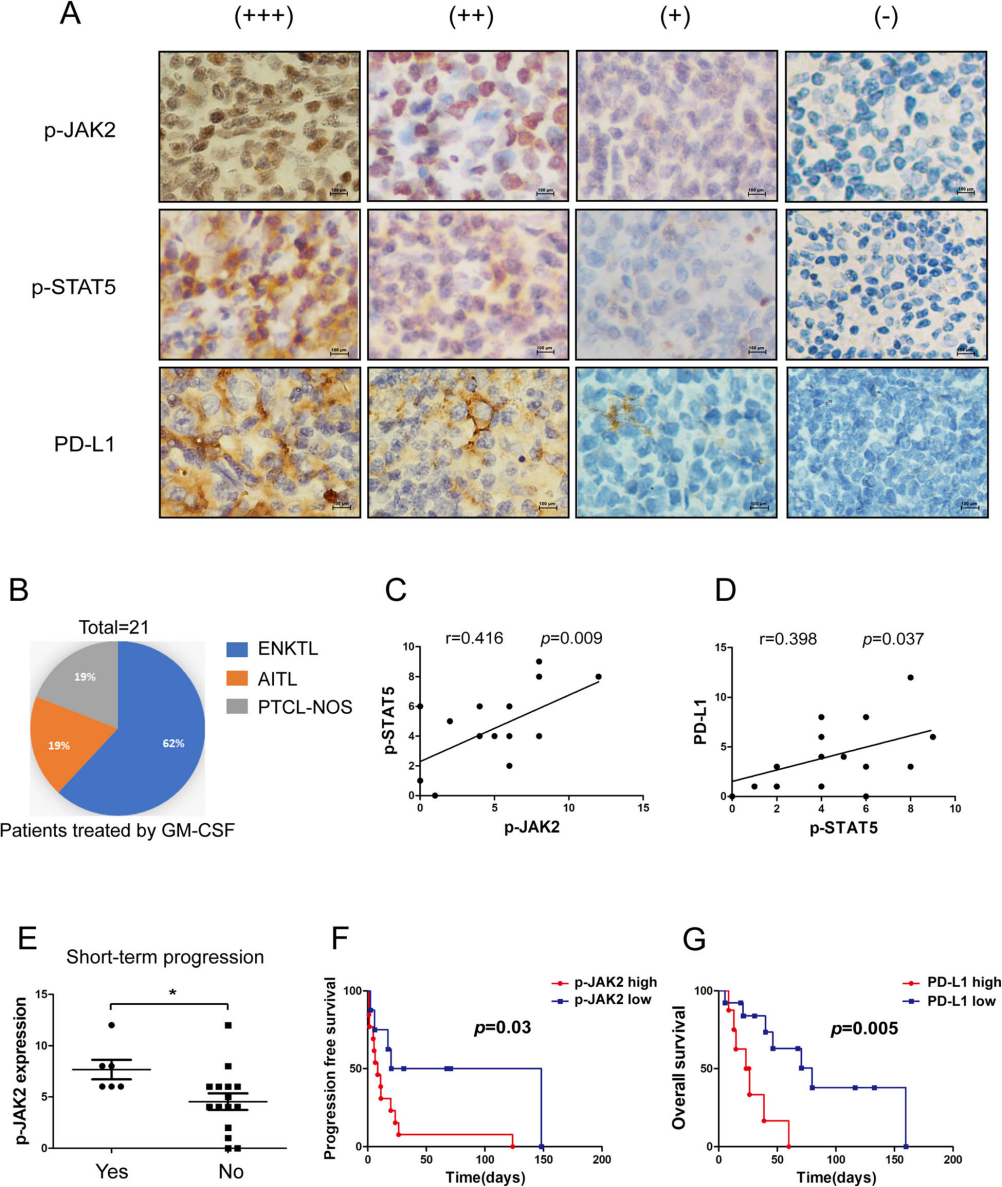
Correlations among p-JAK2, p-STAT5, and PD-L1 expression in ENKTL and PTCL tissues. **a** The representative immunohistochemical stainings of p-JAK2, p-STAT5, and PD-L1 in ENKTL tissues were shown. **b** Percent distribution of GM-CSF treatments for mature T-cell lymphoma. **c** Linear regression analysis of p-JAK2 and p-STAT5 immunohistochemical scores in ENKTL tissue microarray; *P* = 0.009, r = 0.416. **d** Linear regression analysis of p-STAT5 and PD-L1 immunohistochemical scores in ENKTL tissue microarray; *P* = 0.037, r = 0.398. **e** Immunohistochemical scores of p-JAK2 in patients with short-term progression after GM-CSF treatment or not. **f** Kaplan-Meier plots for progression-free survival analysis by the optimal cutoff value of p-JAK2 immunohistochemical scores. Samples were grouped as p-STAT5 high (H-score > 4.5), p-STAT5 low (H-score < 4.5). **g** Kaplan-Meier plots for overall survival analysis by the optimal cutoff value of PD-L1 immunohistochemical scores. Samples were grouped as PD-L1 high (H-score > 5.0), PD-L1 low (H-score < 5.0)

To determine the role of JAK2/STAT5/PD-L1 pathway in the prognosis of ENKTL patients treated by GM-CSF, we used immunostaining to detect the levels of these three proteins in 21 PTCL patients who received GM-CSF treatment from January 2011 to September 2015, including 14 ENKTL patients, 4 AITL patients and 4 PTCL-NOS patients (Fig. 6A-B). We investigated the correlation between the expression of p-JAK2, p-STAT5, and PD-L1 in ENKTL, and found that p-JAK2 had a clear positive correlation with p-STAT5 levels (*p* = 0.001, r = 0.801, Spearman rank correlation coefficient; Fig. 6C). Meanwhile, p-STAT5 had a clear positive correlation with PD-L1 levels (*p* = 0.037, r = 0.398, Spearman rank correlation coefficient (Fig. 6D). Disease progression that developed within a week after GM-CSF treatment was defined as short-term progression. We found the tumor samples from patients with short-term progression, which account for 28.6% (6/21) of all patients, expressed a higher level of p-JAK2 (*p* = 0.019) (Fig. 6E).

We calculated the optimal cutoff point according to ROC curves by comparing the sensitivity and specificity of progression-free survival (PFS) prediction and overall survival (OS). The cutoff expression value of PD-L1 was 5.0 for OS, and the cutoff expression value of p-JAK2 was 5.0 for PFS. Kaplan-Meier curves and log-rank tests were performed. We observed that Patients with low p-JAK2 levels had a longer PFS than those with high p-JAK2 levels (median PFS 25.3 days vs. 8.4 days; *P* = 0.03) (Fig. 6F). Patients with low PD-L1 levels had longer OS than those with high PD-L1 levels (median OS 46.2 days vs. 24.5 days; *P* = 0.005) (Fig. 6G). These data show that high expression of PD-L1 and p-JAK2 both predict poor prognosis of GM-CSF treatment. These data show that high expression of p-JAK2 and PD-L1 both predict inferior prognosis of GM-CSF treatment.

### Somatic alteration of *STAT5A/B* is highly prevalent in ENKTL patients

**Fig. 7:**
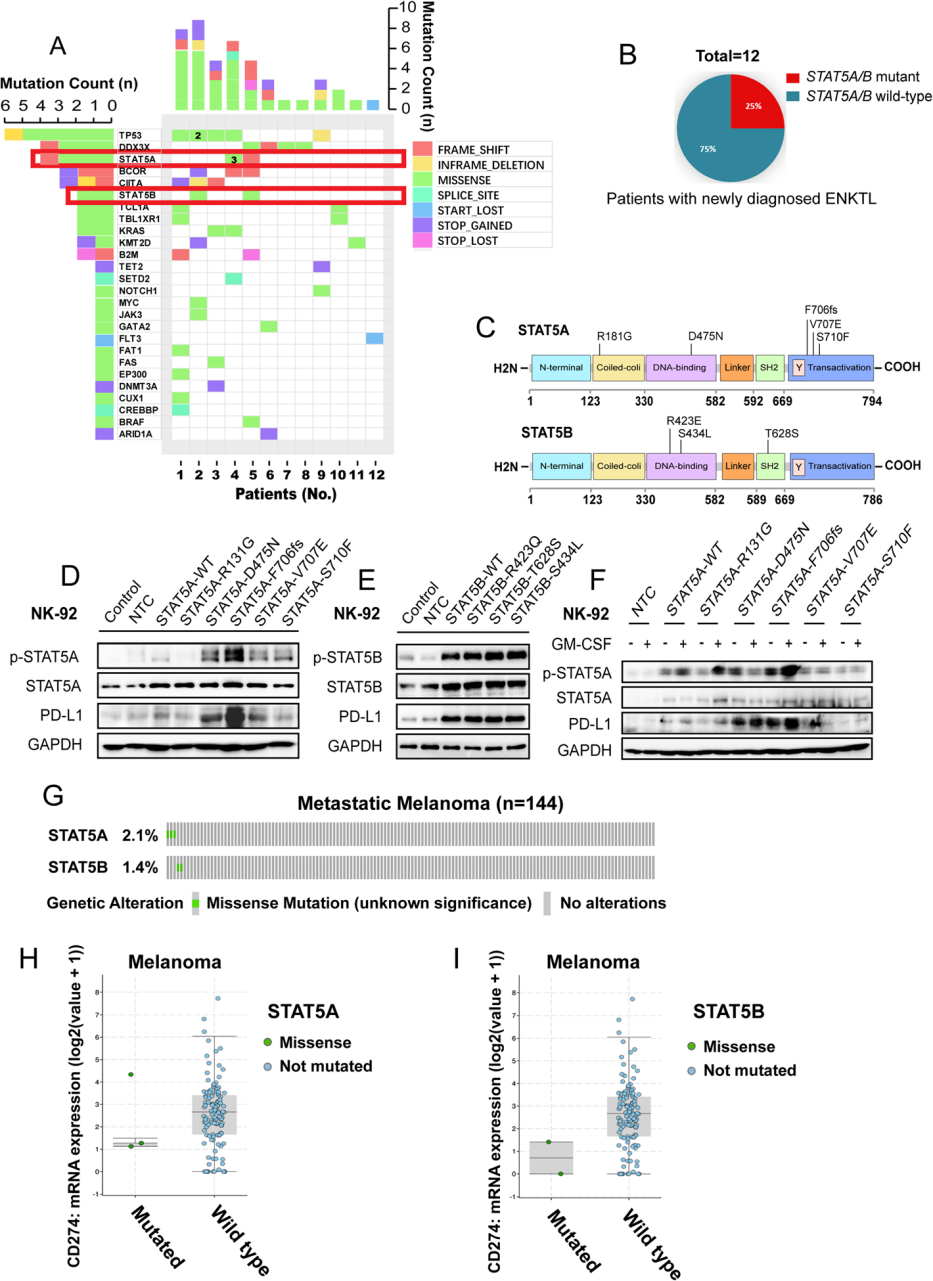
Frequently mutated genes related to ENKTL identified by NGS. **a** The frequency of mutated genes is shown with the last row indicating the first gene with 1 recurrent mutation. Bar (left) represents the number of samples with mutations and bar (top) represents the number of mutations in each sample. **b** Percent of patients who had STAT5A/B mutations. **c** Locations of STAT5A/B mutations in ENKTL. **d** NK-92 cells were transduced with empty vector, STAT5A^WT^, p.R131g, p.D475N, p.F706fs, p.V707E and p.S710F expression vectors. The PD-L1, p-STAT5, and total STAT5 protein levels in these cells were detected with Western blot. **e** NK-92 cells were transduced with empty vector, *STAT5B*^*WT*^, p. R423Q, p.T628S, p.S434L expression vectors. The PD-L1, p-STAT5, and total STAT5 protein levels in these cells were detected with Western blot. **f** NK-92 cells which were transduced with empty vector, *STAT5A*^*WT*^, p.R131G, p.D475N, p.F706fs, p.V707E, and p.S710F expression vectors were treated with GM-CSF (100 ng/ml) for 12 h. The PD-L1, p-STAT5, and total STAT5 protein levels in these cells were detected by Western blot. **g** STAT5A/B mutation rate of 144 melanoma patients reported by David Liu et al. [38]. **h** Scatter plots represent the CD274 mRNA of 144 melanoma patients (STAT5A mutant patients vs. STAT5A wildtype patients). **I** Scatter plots represents the CD274 mRNA of 144 melanoma patients (STAT5B mutant patients vs. STAT5B wildtype patients)

To confirm the role of JAK/STAT pathway in ENKTL, we performed NGS for 102 genes related to ENKTL in 12 cases from January 2018 to March 2019. A total of 47 non-synonymous somatic mutations in 26 genes were identified. Mutations were most frequently located in *STAT5A/B* (12.8%), *TP53* (12.8%), followed by *DDX3X* (8.5%) (Fig. 7A-B). These 47 variants comprised 29 missense, 6 nonsense, 6 frameshift, 2 inframe coding indels, 2 splicing site mutations, 1 missense mutation affecting start codon, and 1 nonstop mutation. A total of 11 genes were found to be recurrently mutated (Fig. 7A). *STAT5A/B* mutations were observed in 25% of cases (3/12), including p.D475N (1/6), p.F706fs (1/6), p.V707E (1/6), p.S710F (1/6) for *STAT5A*, and p.T628S (1/6), p.S434L (1/6) for *STAT5B*. Two mutants of *STAT5A/B* were found in ENKTL cell lines by whole-exome sequencing (WES), including p.R131G for *STAT5A* and p.R423Q for *STAT5B* (Additional file: Table S4). And the melanoma cell line A375 has no mutation of *STAT5A/B* tested by WES (Additional file: Table S4). All of the 8 mutations of *STAT5A/B* were located by cBioPortal (https://www.cbioportal.org/), and the sequences and structures of *STAT5A/B* were described in previous researches (Fig. 7C) [45, 46]. Then we search for the *STAT5A/B* mutations of melanoma patients in cBioPortal, which could visualize the databases of published researches. Five *STAT5A/B* mutations were found in 144 melanoma patients (3.5%) reported by David Liu et al. (2019) (Fig. 7G) [38]. Among the 5 *STAT5A/B* mutant melanoma samples, there has no significant increase in PD-L1 mRNA than *STAT5A/B* wildtype samples (Fig. 7H-I).

**Fig. 8:**
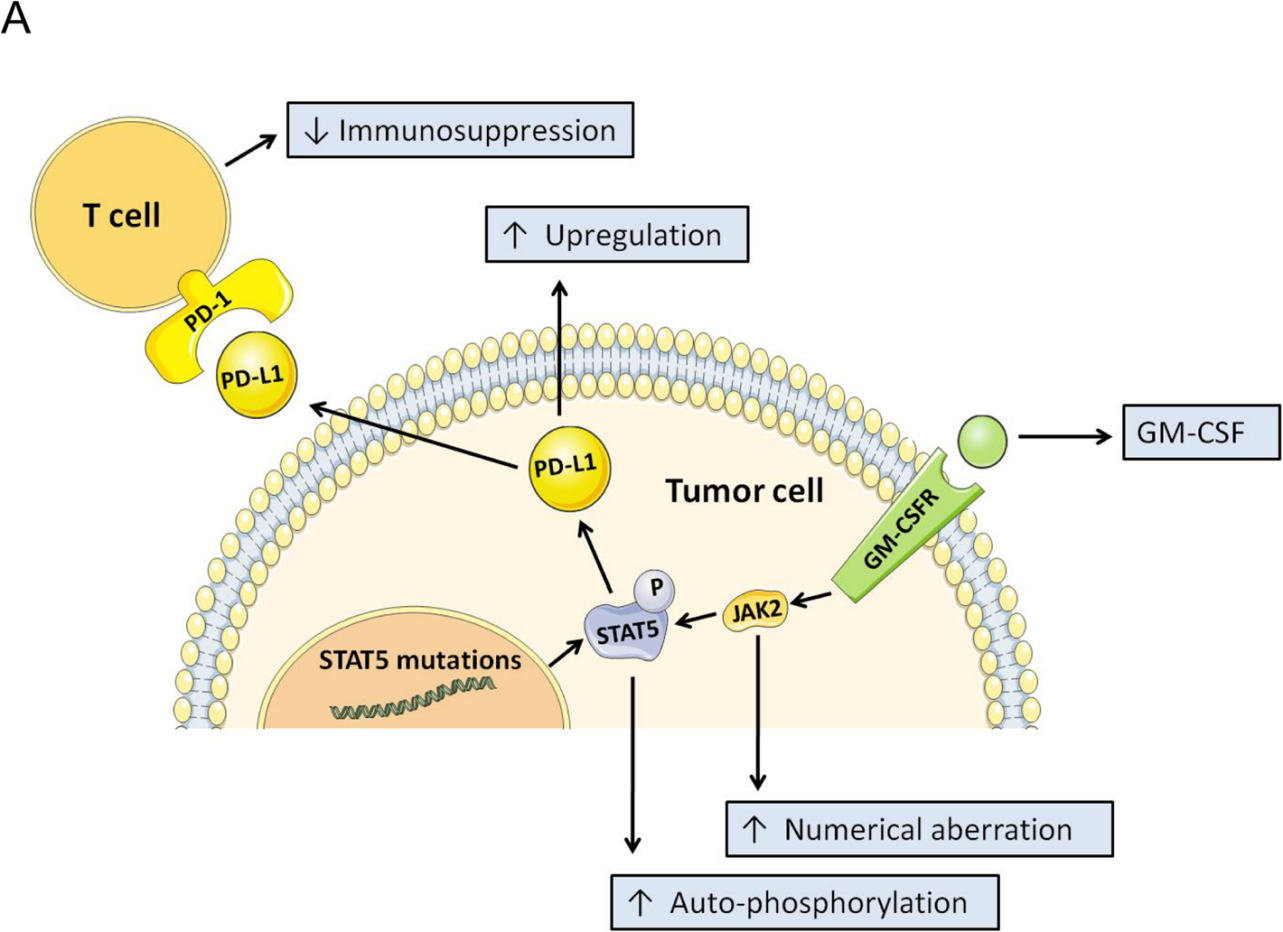
STAT5A mutation increases the PD-L1 overexpression in the presence of GM-CSF. **A** A proposed working model to illustrate how GM-CSF induced disease progression in ENKTL. STAT5 mutations in ENKTL increased the auto-phosphorylation of STAT5. JAK2 hyperphosphorylation combined with STAT5 mutations led to aggressive up-regulation of PD-L1 expression after GM-CSF treatment, which could induce disease progression in ENKTL

To explore the functional implications of these mutations, we generated expression constructs for wild-type, p.D475N, p.F706fs, p.V707E, p.S710F, and p.R131G variants of the STAT5A protein, as well as wild-type, p.T628S, p.S434L, and p.R423Q variants of the STAT5B protein. When expressed in NK-92 cells, increased auto-phosphorylation of STAT5A and PD-L1 has been observed in *STAT5A* mutations (p.D475N and p.V706fs) (Fig. 7D). NK-92 cells harboring p.T628S, p.S434L and p.R423Q vectors have no significant change in auto-phosphorylation of STAT5B and PD-L1 compared to cells harboring *STAT5B*^*WT*^ vector (Fig. 7E). NK-92 cells harboring p.V706fs (c.2118_2119delTG) vector had more aggressive growth of p-STAT5A and PD-L1 than cells harboring *STAT5A*^*WT*^ vectors after GM-CSF treatment (100 ng/ml)(Fig. 7F). The overview of the mechanism for GM-CSF-induced disease progression in ENKTL was shown in Fig. 8A.

## Discussion

GM-CSF was regarded as an immunopotentiator in recent years [47–49], and showed significant and curative effects on several types of tumors when combined with immune checkpoint inhibitors [50, 51], which has an opposite therapeutic effect in ENKTL. Previous studies found that recurrent numerical aberrations of JAK2 in ENKTL, which might be one of the main causes of disease progression induced by GM-CSF [24]. According to our results, the activation of JAK2/STAT5 pathway upregulated the expression of PD-L1 in ENKTL cells and induces persistent immunosuppression status after GM-CSF treatment in vivo. Therefore, GM-CSF might induce disease progression through JAK2/STAT5/PD-L1 axis in ENKTL.

We proved that GM-CSF did not induce the proliferation of ENKTL directly and used different approaches to demonstrate that GM-CSF could upregulate the PD-L1 expression in ENKTL. Furthermore, we found that GM-CSF could induce disease progression in vivo. Mouse treated with GM-CSF have decreased TILs (lower levels of CD3+ and CD8+ T cells than mouse treated with IgG control) and granzyme B from cytotoxic T cells, which could be reversed by anti-PD-1 therapy. The different phenomenon could be seen in melanoma cell line A-375 and B16-F10, which was proved to be a kind of GM-CSF sensitive tumor [50]. We found the number of TILs were decreased in the EL-4 cell bearing tumor mouse treated by GM-CSF, but did not observe the alteration of TIL number in the B16-F10 cell bearing tumor mouse treated by GM-CSF. JAK2 and STAT5 are highly expressed in EL-4 cells but are low expressed in B16-F10 cells. Furthermore, compared with the B16-F10 cells, the expression of PD-L1 was increased in EL-4 cells treated by GM-CSF. Previous researches have proved that high expression of PD-L1 could induce immunosuppression in vivo [52, 53]. These results suggest the decrease of TILs induced by GM-CSF is associated with the expression levels of Jak2 and STAT5 in cancer cells. A previous study has reported that GM-CSF could induce resistance to imatinib and nilotinib in chronic myeloid leukemia (CML) via the activation of JAK2/STAT5, but didn’t mention the Immunological role of JAK2/STAT5 pathway in anti-tumor therapy [54]. GM-CSF-induced PD-L1 expression of neutrophil infiltration in tumors, which could induce immunosuppression in gastric cancer (GC) [21]. We demonstrated that GM-CSF could induce PD-L1 expression in ENKTL cells. But the mechanism of disease progression induced by GM-CSF in ENKTL patients still needs to be further explored due to the lack of comparable tumor samples of patients after GM-CSF treatment and limitation of the small sample size.

A recent study has reported that alterations in JAK/STAT pathway is highly prevalent in ENKTL, and STAT3 was one of the most frequently mutated in exons of 188 JAK/STAT pathway-related genes. They proved that the frequent mutations of STAT3 might be one of the reasons for high PD-L1 expression in ENKTL [55]. We used NGS with a different range of panels that targeted a total of 102 ENKTL-relevant genes. We also found that *STAT5A/B* mutations are highly prevalent in ENKTL, and *STAT5A* was one of the most frequently mutated genes in ENKTL patients. Early Research has reported that *STAT5* mutations are rare in hematopoietic diseases [56], and remained few studies about *STAT5* mutations in lymphoma so far. Therefore, exploration of PD-L1 expression regulated by *STAT5* mutations, especially in ENKTL, is still meaningful in the future. Cells harboring p.D475N and p.V706fs (c.2118_2119delTG) vectors had increased auto-phosphorylation of STAT5A and PD-L1. More importantly, cells harboring p.V706fs (c.2118_2119delTG) vector had more aggressive growth of p-STAT5A and PD-L1 expression, which might promote tumor immune evasion. We thought frequent *STAT5A* mutations and elevated expression of JAK2 might be an enhancer for generous up-regulation of PD-L1 expression induced by GM-CSF in ENKTL.

These findings may support the phenomenon that ENKTL patients have the effect as opposed to patients with other types of tumor after the treatment of GM-CSF. Furthermore, we hypothesized that GM-CSF would lose its ability to induce immunosuppression and carry out its function as an immunopotentiator after blocking the PD-1/PD-L1 pathway. The hypothesis may produce a better explanation for the result that mouse-bearing EL4 tumors treated by GM-CSF combined with anti-PD-1 therapy had enhanced secretion of granzyme B compared to those mice treated with anti-PD-1 therapy alone. A previous study has proved that GM-CSF alone may limit the limit anti-tumor immunity [57–59], which could be reversed with the addition of checkpoint inhibitor [50]. This may also support the clinical investigation in the combination of GM-CSF and anti-PD-1 therapy in ENKTL.

In conclusion, our research demonstrated that GM-CSF potentially triggered the loss of tumor immune surveillance in ENKTL patients and promoted disease progression, which was associated with STAT5A mutations and JAK2 hyperphosphorylation, and then upregulates the expression of PD-L1. These may provide new concepts for GM-CSF application and new strategies for the treatment of ENKTL.

## Supplementary Information

**Additional file 1: Table S1.** 102-gene list for targeted next-generation sequencing.

**Additional file 2: Table S2.** Clinical characteristics.

**Additional file 3: Table S3.** Univariate analysis of prognostic factors for survival.

**Additional file 4: Table S4.** Whole exome sequencing (WES).

## Data Availability

The data generated and analyzed will be made from the corresponding author on reasonable request. The authenticity of this article has been validated by uploading the key raw data onto the Research Data Deposit public platform with an RDD number of RDDB2021000972 (www.researchdata.org.cn).
